# crosshap: R package for local haplotype visualization for trait association analysis

**DOI:** 10.1093/bioinformatics/btad518

**Published:** 2023-08-22

**Authors:** Jacob I Marsh, Jakob Petereit, Brady A Johnston, Philipp E Bayer, Cassandria G Tay Fernandez, Hawlader A Al-Mamun, Jacqueline Batley, David Edwards

**Affiliations:** Centre for Applied Bioinformatics, University of Western Australia, Perth WA, 6009, Australia; School of Biological Sciences, University of Western Australia, Perth WA, 6009, Australia; Centre for Applied Bioinformatics, University of Western Australia, Perth WA, 6009, Australia; School of Biological Sciences, University of Western Australia, Perth WA, 6009, Australia; School of Molecular Sciences, University of Western Australia, Perth WA, 6009, Australia; Centre for Applied Bioinformatics, University of Western Australia, Perth WA, 6009, Australia; School of Biological Sciences, University of Western Australia, Perth WA, 6009, Australia; Centre for Applied Bioinformatics, University of Western Australia, Perth WA, 6009, Australia; School of Biological Sciences, University of Western Australia, Perth WA, 6009, Australia; Centre for Applied Bioinformatics, University of Western Australia, Perth WA, 6009, Australia; School of Biological Sciences, University of Western Australia, Perth WA, 6009, Australia; Centre for Applied Bioinformatics, University of Western Australia, Perth WA, 6009, Australia; School of Biological Sciences, University of Western Australia, Perth WA, 6009, Australia; Centre for Applied Bioinformatics, University of Western Australia, Perth WA, 6009, Australia; School of Biological Sciences, University of Western Australia, Perth WA, 6009, Australia

## Abstract

**Summary:**

Genome-wide association studies (GWAS) excels at harnessing dense genomic variant datasets to identify candidate regions responsible for producing a given phenotype. However, GWAS and traditional fine-mapping methods do not provide insight into the complex local landscape of linkage that contains and has been shaped by the causal variant(s). Here, we present crosshap, an R package that performs robust density-based clustering of variants based on their linkage profiles to capture haplotype structures in a local genomic region of interest. Following this, crosshap is equipped with visualization tools for choosing optimal clustering parameters (ɛ) before producing an intuitive figure that provides an overview of the complex relationships between linked variants, haplotype combinations, phenotype, and metadata traits.

**Availability and implementation:**

The crosshap package is freely available under the MIT license and can be downloaded directly from CRAN with *R* >4.0.0. The development version is available on GitHub alongside issue support (https://github.com/jacobimarsh/crosshap). Tutorial vignettes and documentation are available (https://jacobimarsh.github.io/crosshap/).

## 1 Introduction

Rapidly accumulating genomic sequence information presents opportunities to identify and characterize the effects of variants conferring beneficial adaptations. However, with increasing population sizes, the dimensions of variant information increase exponentially, raising interpretability issues when analysing large genomic datasets. Genome-wide association studies (GWAS) offer a scalable approach to identify candidate regions responsible for influencing a phenotype, though they do not provide insight into the complex local landscape of linkage that contains and, in many cases, has been shaped by selection for the causal variant(s).

Statistical fine-mapping is the standard approach for visualizing phenotypic associations for local genomic variants surrounding a GWAS result (Schaid *et al.* 2018). These methods excel at isolating candidate variants responsible for simple traits with strong phenotypic associations caused by explainable genomic changes such as nonsense mutations (Pruim *et al.* 2010). However, fine-mapping only facilitates users to evaluate variants in a region of interest by unidimensional phenotypic association scores, and linkage relationships between variants are only represented by simple summary statistics (Marsh *et al.* 2022). In addition, fine-mapping visualization tools do not link analysis of genomic variation with metadata features of individuals across the population, such as level of domestication or region collected, which may be important co-factors influencing phenotypic association scores.

Local haplotyping defines groups of individuals based on allelic combinations across loci in a region of interest (Belzile *et al.* 2020). The primary advantage of this approach over statistical fine-mapping is that it connects shared genomic variation to the individual, which aids in capturing the phenotypic effects of haplotypes, and allows for characterization of the identified populations that possess shared allelic combinations within the region of interest (Marsh *et al.* 2022). The rice-specific RFGB v2.0 (Wang *et al.* 2019) and the Sanger-sequencing compatible CandiHap (Li *et al.* 2023) are allele mining tools which capture local haplotypes as unique combinations of all single nucleotide polymorphisms (SNPs) in a window centred on a gene of interest. However, when a trait is associated with a larger region that cannot be reduced to a single gene and instead spans an interval with dozens, hundreds or thousands of variant loci, minor divergences between individuals result in a rapid expansion of the number of unique haplotypes identified by these methods, restricting effective analysis between groups.

LD-based local haplotyping approaches such as HaplotypeMiner (Tardivel *et al.* 2019) enable analysis of wider windows through variant dimension reduction, with ‘tagging’ to prune redundant SNPs in high linkage disequilibrium (LD) before defining haplotypes. Alternatively, HapFM (Wu *et al.* 2022) is an end-to-end causal candidate haplotype identification tool that clusters individuals into haplotypes from local variation to perform an improved GWAS. The strengths of HaplotypeMiner and HapFM lie in their ability to synthesize general patterns of linkage from large complex variant data to assign individuals into relevant groups for downstream analysis. However, current local haplotyping software is limited in its ability to convey the relevance of haplotyping results to the user. For example, HaplotypeMiner does not integrate phenotype data with haplotypes, and HapFM currently does not visualize results, often requiring new users to manually develop their own downstream analysis pipelines to effectively interpret the output from these tools (see Supplementary Note 1 for comparison of local haplotyping tools). Realizing the full potential of LD-based local haplotyping requires drawing connections between the groups of linked variants/haplotype combinations identified and meaningful phenotype and metadata differences between individuals. Informative genomic visualizations are needed to de-convolute complex linkage structures by displaying the relationships between variants within and between each Marker Group whilst also connecting both Marker Group and haplotype combination results back to individual-level differences.

Here, we present crosshap, which performs robust density-based clustering of variants based on their linkage profiles to capture haplotype structures in local genomic regions. crosshap supports highly transparent haplotyping, equipping the user with a wide range of tools to understand patterns of local genomic variation through curated visualizations displaying raw data points. Visualization tools are provided by crosshap for choosing optimal clustering parameters (ɛ) and producing intuitive crosshap figures that present information on the complex relationships between linked variants, haplotype combinations, and phenotype and metadata traits of individuals.

## 2 Main

**Figure 1.**
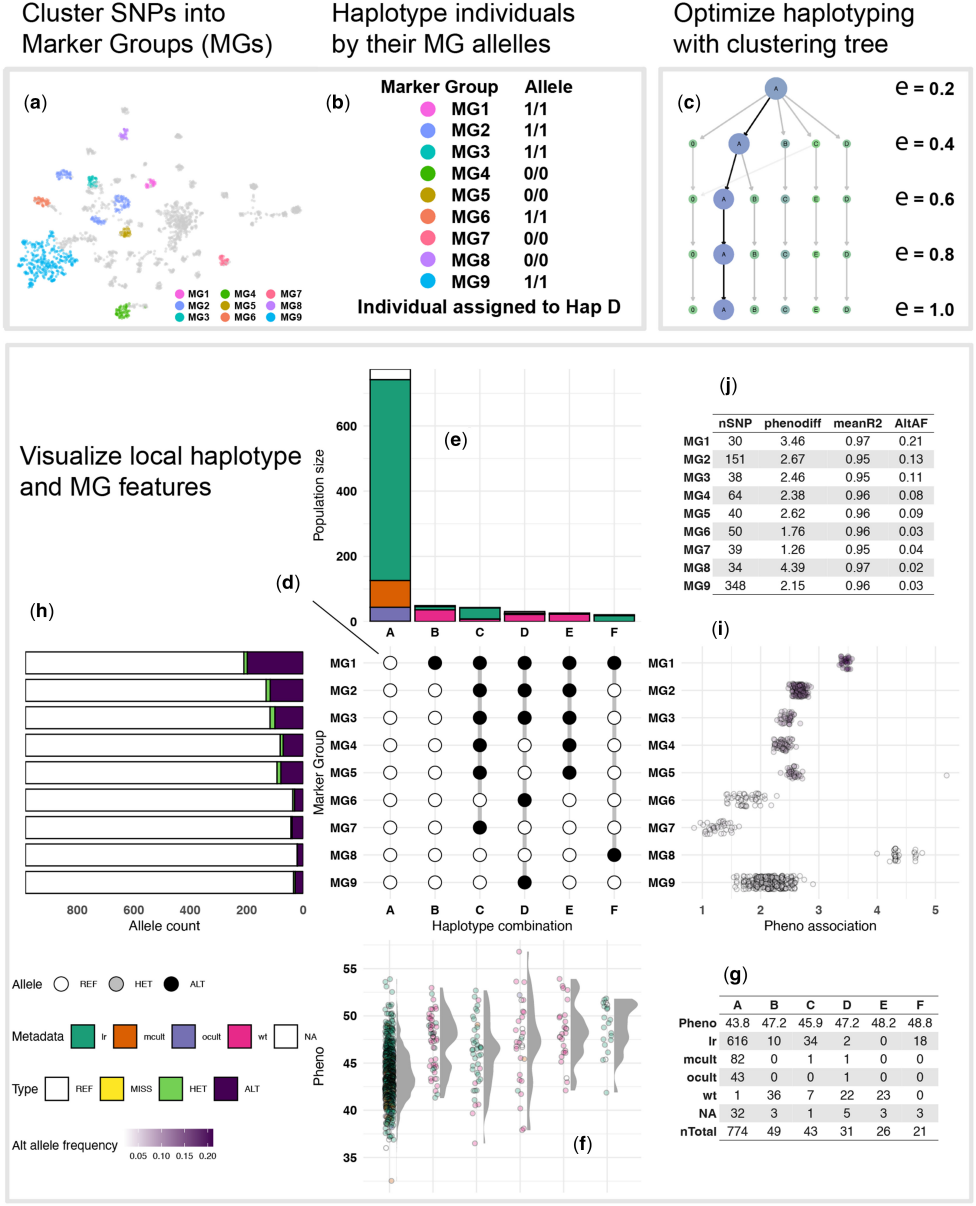
Overview of the local haplotype analysis pipeline performed by the three core ‘crosshap’ functions. run_haplotyping performs Marker Group generation, represented by a UMAP of all SNPs co-located based on linkage, coloured by assigned Marker Group (a), as well as haplotyping of individuals (b), before returning haplotype objects with results. clustree_viz generates a clustering tree (c) to aid users in choosing an optimal epsilon. crosshap_viz generates a comprehensive visualization of a haplotyping results (d–j). The Marker Group alleles defining each haplotype are represented in the centre (d), results related to individuals and haplotypes are visualized vertically (e–g), results related to Marker Groups and SNPs are visualized horizontally (h–j).

The crosshap workflow is outlined in Fig. 1. For haplotyping, crosshap requires genomic variant information in VCF format, delimited to a region of interest (see https://jacobimarsh.github.io/crosshap/articles/Delimiting_region.html), along with a square matrix reporting a pairwise LD statistic (e.g. *R*^2^) between all loci. Phenotype and metadata are two additional input classes describing features of the individuals in the VCF that aid visualization and interpretation of haplotyping results. Phenotype supports any numerical description of individuals, such as yield or time to maturity. Metadata, which is optional input, supports any categorical description of individuals, such as subspecies or region collected.

### 2.1 Defining haplotypes

The first stage of local haplotyping with crosshap is clustering of SNPs into representative ‘Marker Groups’ (MGs) using density-based spatial clustering of applications with noise (DBSCAN) (Ester *et al.* 1996) (see Supplementary Note 3 for discussion of alternative variant clustering algorithms). Following this, the most common (arithmetic mode) allelic states (homozygous reference/homozygous alternate/heterozygous) across the SNPs within each Marker Group are calculated for each individual, which in turn becomes their assigned allele for the entire Marker Group (Fig. 1a).

Following the assignment of Marker Group allelic states for each individual, the frequency of each unique allelic combination is reported. Haplotype combinations with a minimum frequency of individuals (default minHap = 9) are assigned a unique label (A–Z) and kept for downstream analysis. Marker Groups that are invariant across the haplotype combinations are removed, and the remaining Marker Groups are provided with the prefix ‘MG’ and an identifying number (e.g. ‘MG1’). Individuals sharing MG allele combinations are assigned to a haplotype group (Fig. 1b). run_haplotyping returns a haplotype object containing results for several user-provided epsilon values.

### 2.2 Optimization with clustering tree

To aid users in choosing an optimal epsilon (controls number and size of MGs/haplotypes) for DBSCAN clustering of variants, crosshap provides a clustree (Zappia and Oshlack 2018) wrapper that summarizes differences between MGs and haplotype populations identified at different epsilon levels in a clustering tree (Fig. 1c). The clustering tree fulfils the same role as the commonly used k-nearest neighbour (KNN) distance plot (Kriegel *et al.* 2011) by displaying cluster stability at different epsilon values. However, it is better suited to optimizing variant dimension reduction for local haplotyping because a clustering tree isolates the effects of epsilon changes on each individual cluster rather than reflecting the mean KNN distance across all points, which is sensitive to outliers. In addition to displaying cluster stability and size (nSNP or nIndividuals), the crosshap clustering tree indicates the phenotypic association of each MG or haplotype population.

### 2.3 Visualizing haplotypes

The crosshap visualization provides users with a comprehensive dashboard incorporating several plots to intuitively connect MG features with haplotype combination attributes (Fig. 1d–j). This allows users to quickly identify which individuals or demographic group frequently possess specific MG alleles, providing insight into the patterns of genomic variability across populations.

Local haplotype visualization with crosshap is anchored around a central matrix that depicts the MG allelic combinations defining each haplotype group (Fig. 1d), modelled from the UpSet plot approach of visualizing intersecting sets (Lex *et al.* 2014). This can be useful to identify nested linkage patterns, where specific MG may be shared between haplotypes that differ across other variants. Surrounding the central matrix are peripheral plots, with attributes of the individuals in each haplotype displayed vertically in the top and bottom plots (Fig. 1e and f). Across each haplotype, their frequency, phenotype scores and spread of individuals from different demographic groups are captured (Fig. 1e and f), with additional functionality to isolate and report the phenotype association of specific demographic groups with the isolate_groups option.

Attributes of SNPs in each MG are displayed horizontally in the left and right plots (Fig. 1h and i). The frequencies of the different allelic states are captured as well as the phenotype association. The phenotype association of SNPs is calculated as the difference between individuals with reference and alternate alleles, assuming additive effects for heterozygous individuals (i.e. phenotype scores from homozygous individuals are weighted double those from heterozygous individuals). Alternative plots are provided to users with the plot_left and plot_right switches, which can be used to instead display the relative genomic positions of SNPs within each MG across the region of interest, and a boxplot that represents the extent and sphericity of intra-MG linkage. Additional tabular summaries are provided to supplement the plots with haplotype information in the bottom-left (Fig. 1g) and SNP information in the top-right (Fig. 1j).

Detailed vignettes are provided alongside documentation to enable users to prepare inputs, learn the core functionality of crosshap, and build a UMAP GIF visualization to aid in conceptualizing the variation captured by local haplotyping at https://jacobimarsh.github.io/crosshap/.

## 3 Conclusion and perspectives

The primary use-case of crosshap is as a follow-up to GWAS or QTL mapping, to elucidate patterns of inheritance in a trait-associated interval of interest. Visualization with crosshap aids in characterizing genomic variation in the region and is valuable for uncovering novel features and relationships between haplotypes. Following this, clusters of SNPs defined in MGs and the haplotype assignments of individuals become useful for enabling downstream population genetics analysis between relevant groups.

The development of crosshap represents a major advance in the accessibility of reproducible local haplotyping tools. With a focus on guiding parameter optimization and intuitive visualizations that connect genomic variation to individual-level traits, crosshap makes robust local haplotyping accessible to users with limited domain knowledge, equipping users with the tools necessary to develop a deep understanding of linkage patterns in a genomic region of interest.

As trait mapping and association analysis moves towards the widespread use of unsupervised learning approaches with ‘black box’ issues of interpretability, it will be increasingly necessary to follow up on these results by effectively visualizing patterns of local genomic variation to translate the results to the user. Capturing complex linkage structures and local haplotype combinations is an essential component for understanding the inheritance of variants in a genomic region of interest, a task that is made more accessible with the development of crosshap.

## Supplementary Material

btad518_Supplementary_Data

## Data Availability

Data used to generate Fig. 1 is available at figshare (https://figshare.com/articles/dataset/fin_b51_173kb_only_vcf/22331167) and is made available as example data within the ‘crosshap’ package.
