# Supplementary material for: crosshap: R package for local haplotype visualization for trait association analysis
Source: Bioinformatics. 2023 Aug 22;39(8):btad518. doi: 10.1093/bioinformatics/btad518 (PMC10471896; doi:10.1093/bioinformatics/btad518)
Supplement: btad518_Supplementary_Data [file btad518_supplementary_data.docx]

# Supplementary Note 1: Comparisons with other local haplotyping software

**Supplementary Table 1.** Comparison of local haplotyping tools.

|  | HaplotypeMiner | HapFM | RFGB v2.0 | CandiHap | crosshap |
| --- | --- | --- | --- | --- | --- |
| Availability | GitHub package | Scripts only | Web browser* (limited to rice 3K dataset*) | GitHub package | CRAN package |
| Reference | (Tardivel et al., 2019) | (Wu et al., 2022) | (Wang et al., 2019) | (Li et al., 2020) | This publication |
| Haplotyping approach^1^ | LD-based dimension reduction | LD-based dimension reduction | Precise | Precise | LD-based dimension reduction |
| SNP dimension reduction | Representative SNPs through tagging | Haplotype blocking* (Individuals grouped by X-means clustering*) | No | No | Density-based spatial clustering with noise |
| Optimization tools | Pairwise R^2^ between all SNPs at single resolution (Supplementary Figure 1) | No | No | No | Clustering tree wrapper across haplotype resolutions |
| Phenotype | None | Haplotypes only | Haplotypes only | Haplotypes only | Markers and haplotypes |
| Metadata | No | No | No | No | Haplotypes only |
| InDels | No | Yes | No | Yes | No |
| Annotation | No | Yes | Yes | Yes | No |
| Visualizations show unmodified data^2^ | No | No | No | Individuals only | Yes |
| Visualizations connecting haplotypes to markers | Yes (Supplementary Figure 2) | No | No | No | Yes (Figure 1d-j) |

^1^ LD-based dimension reduction refers to methods that group SNPs to reduce redundancy before defining haplotypes, allowing larger windows of SNPs to be characterised. Precise refers to methods that use all SNPs in a local region of interest (e.g. within and surrounding a gene) are used to define haplotypes, which is suitable only for few SNPs in a small window.

^2^ Showing precise features such as phenotype scores individuals and allele frequencies of individual SNPs, rather than composite statistics such as mean phenotype score of haplotypes which can be sensitive to outliers and population structure.

### HaplotypeMiner: approach and optimization

The most similar tool to crosshap in terms of local haplotyping methods and figure generation is HaplotypeMiner (Tardivel et al., 2019). HaplotypeMiner systematically filters and prunes SNPs across a genomic window to dramatically reduce variant dimensionality and identify representative markers based on user-defined parameters. Unique allelic combinations of representative markers are used to define different haplotypes.

Crosshap delivers several key improvements in accessibility and transparency over this method (Supplementary Table 1), most importantly with advanced visualizations. Firstly, LD-based local haplotyping tools such as HaplotypeMiner and crosshap require the optimization of several parameters to generate effective results for a given region using a specific dataset. HaplotypeMiner users must primarily rely on trial and error to find suitable parameters (particularly for the clustering threshold), with some minor assistance provided by a pairwise LD plot that may in some cases aid in selecting a marker independence threshold (Supplementary Figure 1). In contrast, crosshap is designed to run a range of clustering parameters, before providing a detailed clustering tree wrapper (Figure 1c) which summarizes the difference between the haplotyping results, with reference to phenotype data.

See the ‘Optimizing the clustering resolution’ section of crosshap’s ‘Getting Started’ vignette for an example figure and information for how the clustering tree can be interpreted with crosshap (<https://jacobimarsh.github.io/crosshap/articles/Getting_started.html#optimizing-the-clustering-resolution>).

In addition to the clustering tree, crosshap saves several haplotyping results in a single object, allowing users to quickly switch between the complete visualization of haplotypes at different resolutions by simply changing the epsilon parameter passed to `haplotype_viz`.


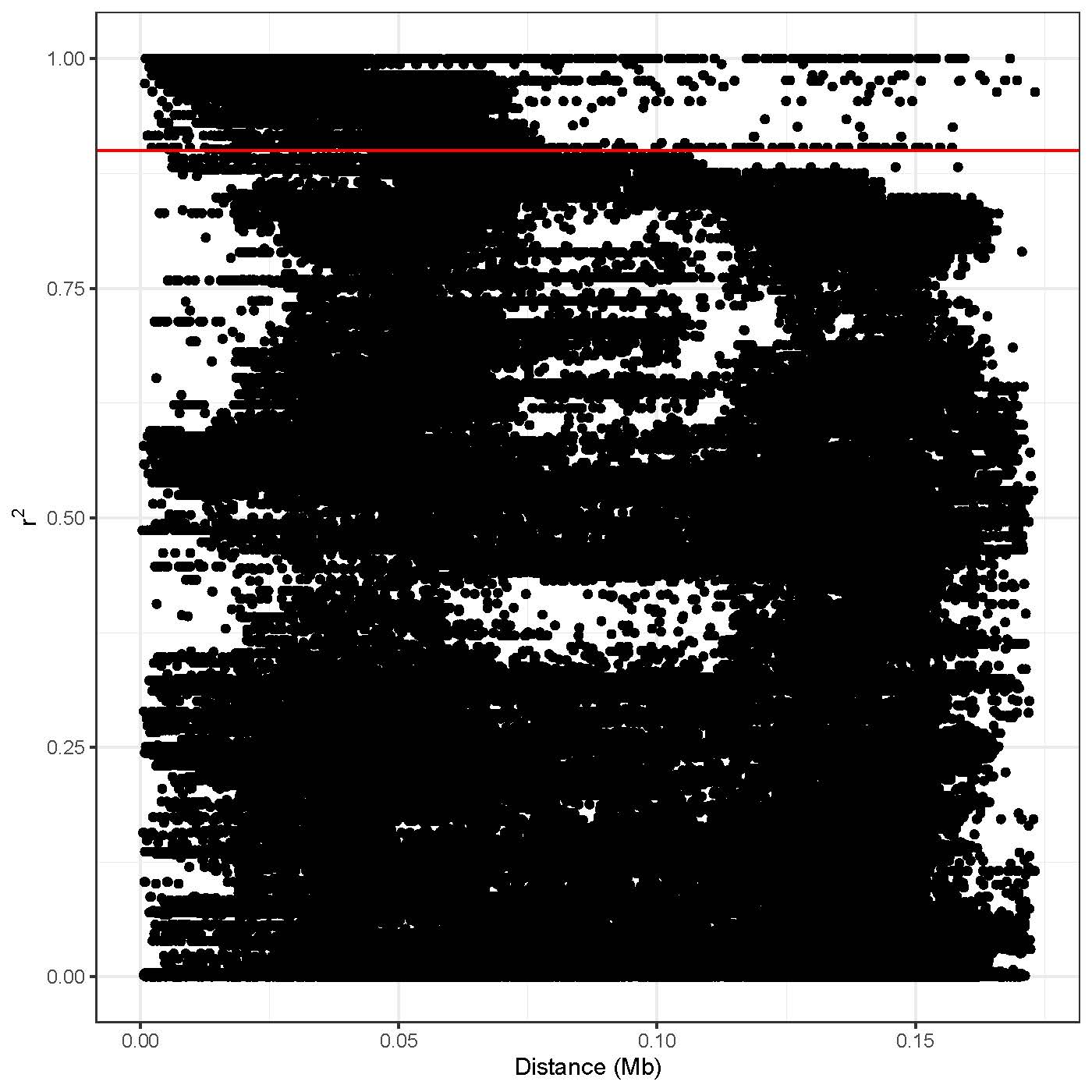


**Supplementary Figure 1.** Estimation of linkage disequilibrium (R^2^) between all pairs of markers across the 173kb region as a function of pairwise distance, generated by HaplotypeMiner to aid with optimizing the marker independence threshold (Tardivel et al. 2019). The red line indicates the final marker independence threshold used for analysis of the cqProt-003 region in Marsh et al (2022). Figure taken directly from Marsh et al. (2022) with permission.

### HaplotypeMiner: Haplotype visualization

The haplotype plot output of HaplotypeMiner provides a table of the combinations of genetic (representative) markers that each haplotype population shares (Supplementary Figure 2), fulfilling the same role as the crosshap visualization (Figure 1d-f), though with several differences. First, HaplotypeMiner uses a greedy pruning algorithm to remove SNPs in high linkage, therefore one specific ‘Marker’ may be representing hundreds of SNPs similar to a Marker Group (see Supplementary Table S5 in Marsh et al. 2022), though it is not clear from the visualization how many SNPs each marker represents, where they are located or to what extent they are lined. In contrast, crosshap explicitly clusters SNPs to remove the bias of only representing a single arbitrarily chosen locus.


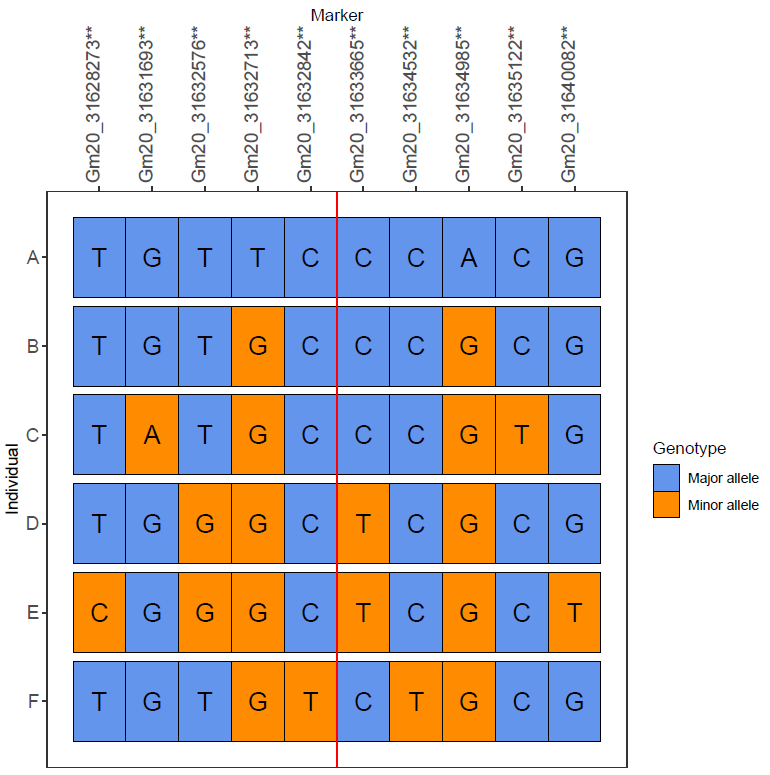


**Supplementary Figure 2.** Plot of haplotypes as combinations of representative markers in local region, generated by HaplotypeMiner (Tardivel et al. 2018) to analyse the *cqProt-003* region in Marsh et al. (2022). Note: ‘Individual’ refers to the different haplotypes, A-F.

The primary, and most important distinction between the HaplotypeMiner haplotype plot and crosshap’s haplotype visualization is the characterization of the defined haplotype and Marker Groups. Crosshap simultaneously displays features such as the frequency of each haplotype, their frequencies across metadata groups (e.g. individuals with level of domestication) and the phenotype scores of individuals with each haplotype. In addition, crosshap characterises the Marker Groups and the SNPs within each Marker Group, with plots indicating minor allele frequencies, missingness/heterozygous allele proportions, position throughout the region of interest, extent of linkage within the cluster, and the associations of all SNPs with a given phenotypic trait.

See the ‘Visualizing local haplotypes’ and the ‘Interpreting the crosshap visualization’ sections of crosshap’s ‘Getting Started’ vignette for an example figure and more information on what’s captured by, and how to interpret the crosshap visualization (https://jacobimarsh.github.io/crosshap/articles/Getting_started.html#visualizing-local-haplotypes).

### HapFM

HapFM is an end-to-end causal candidate haplotype identification tool that delimits a flanking window around a site of interest from haplotype block boundaries (Wu et al., 2022). Following this, X-means clustering is applied to group individuals with similar haplotypes within the identified block boundary. The clusters identified represent groups with relatively distinct haplotypes in the region of interest which are used to provide phenotypic effect scores for each block, aiding in prioritization.

It is not possible to directly compare HapFM and crosshap as they serve two distinct purposes. While HapFM performs local haplotyping, the results are used for association analysis only, providing no framework to further understand or visualize what haplotype combinations may be contributing to the significance of the region. Crosshap in contrast requires a pre-identified region of interest from which it can visualize linkage and association features of variants, variant combinations (haplotypes) and individuals possessing those haplotypes. Therefore, HapFM and crosshap are highly complementary approaches, where HapFM may be used to identify and delimit regions with trait-associated haplotypes which can then be used as input to crosshap for detailed visualization to better understand the features of haplotypes in the region of interest.

# Supplementary Note 2: Example crosshap analysis using HapFM results

Here, we will be briefly demonstrate how an identified trait-associated interval from HapFM can be used as input for further analysis with crosshap. Wu et al. (2022) demonstrate HapFM with two empirical datasets, including *Arabidopsis* *thaliana* data with the flowering time phenotype from the 1001 Genomes project (<https://1001genomes.org>). One of the final QTLs identified by HapFM-anno is Chr1@25408933–25429985 which is a 21kb interval containing 1990 SNPs. We downloaded the same SNP data, metadata information and phenotype data from <https://1001genomes.org> to conduct local haplotype analysis on the 21kb region with crosshap. The SNP data was imputed using Beagle 5.4 (Browning et al., 2021), however no other filtering or pruning was performed.

The region delimited VCF was used as input for crosshap, with an R^2^ LD matrix generated by PLINK (Purcell et al., 2007), the flowering time phenotype data, and country of collection for each sample as metadata (only the 6 most frequent countries included). Parameters passed to crosshap were as follows: `epsilon = c(0,0.6,1,1.3,1.6,2), MGmin = 20`. The clustering tree (Supplementary Figure 3) for the marker groups indicated gradually increasing granularity as epsilon increased, though the Marker Groups MG1 and MG3 (at epsilon = 1), remain highly stable across epsilon resolutions, indicating high linkage among SNPs at these Marker Groups. An epsilon of 1 was chosen for further visualization.


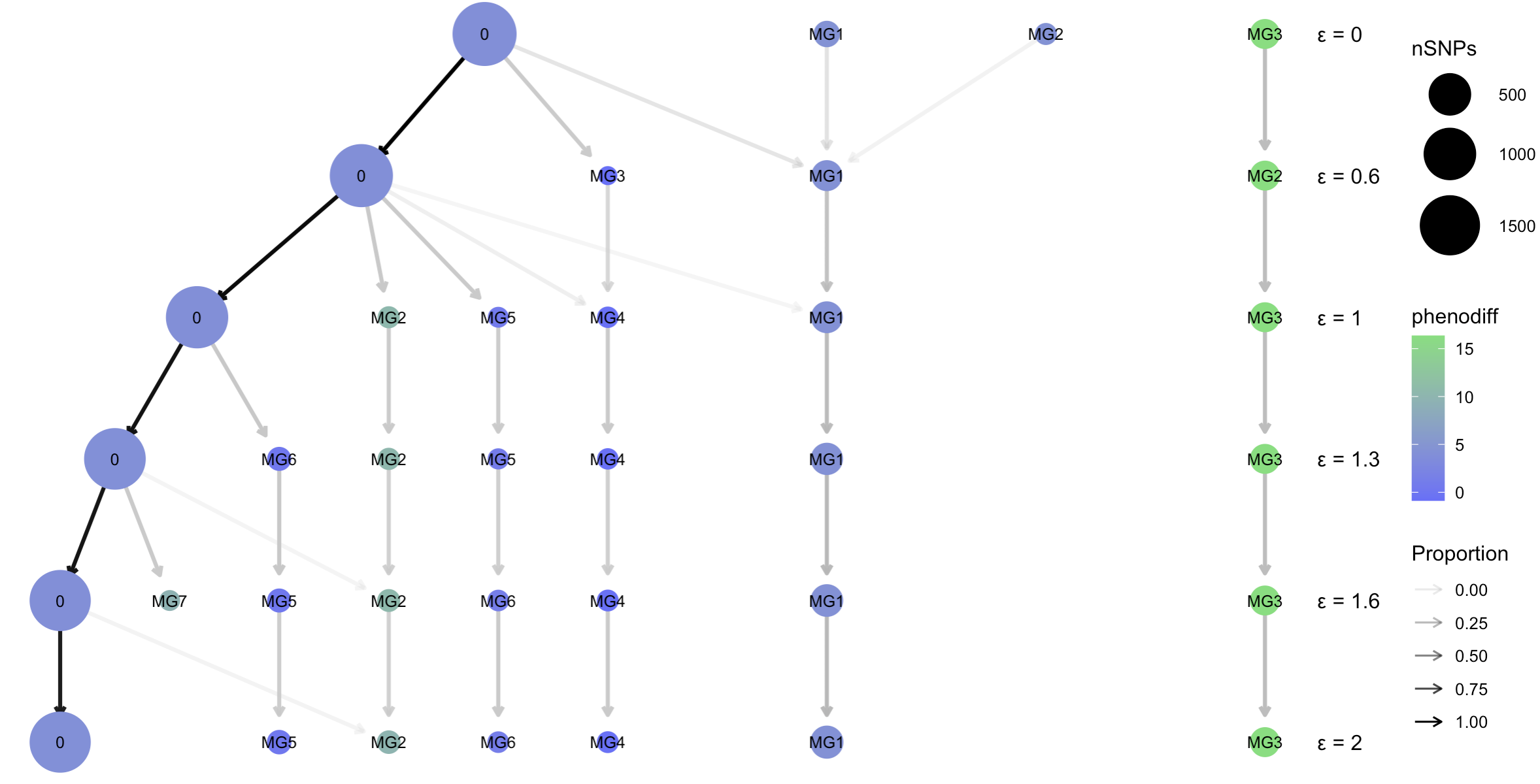


**Supplementary Figure 3.** Clustering tree of Chr1@25408933–25429985 using the *A. thaliana* 1001 Genomes data. The clustering tree depicts changes in marker group clusters resolved at different epsilon values (minPts = 20). The nodes in each row represent the marker groups identified at a single epsilon, with size reflecting the number of SNPs in a marker group, and colour reflecting the mean phenotypic association of all SNPs within a given marker group. The arrows indicate overlaps between the SNPs in two clusters at different epsilon resolutions, with the opacity of the arrow reflecting the proportion of SNPs in the preceding (higher epsilon) node that are assigned to the destination (lower epsilon) node.


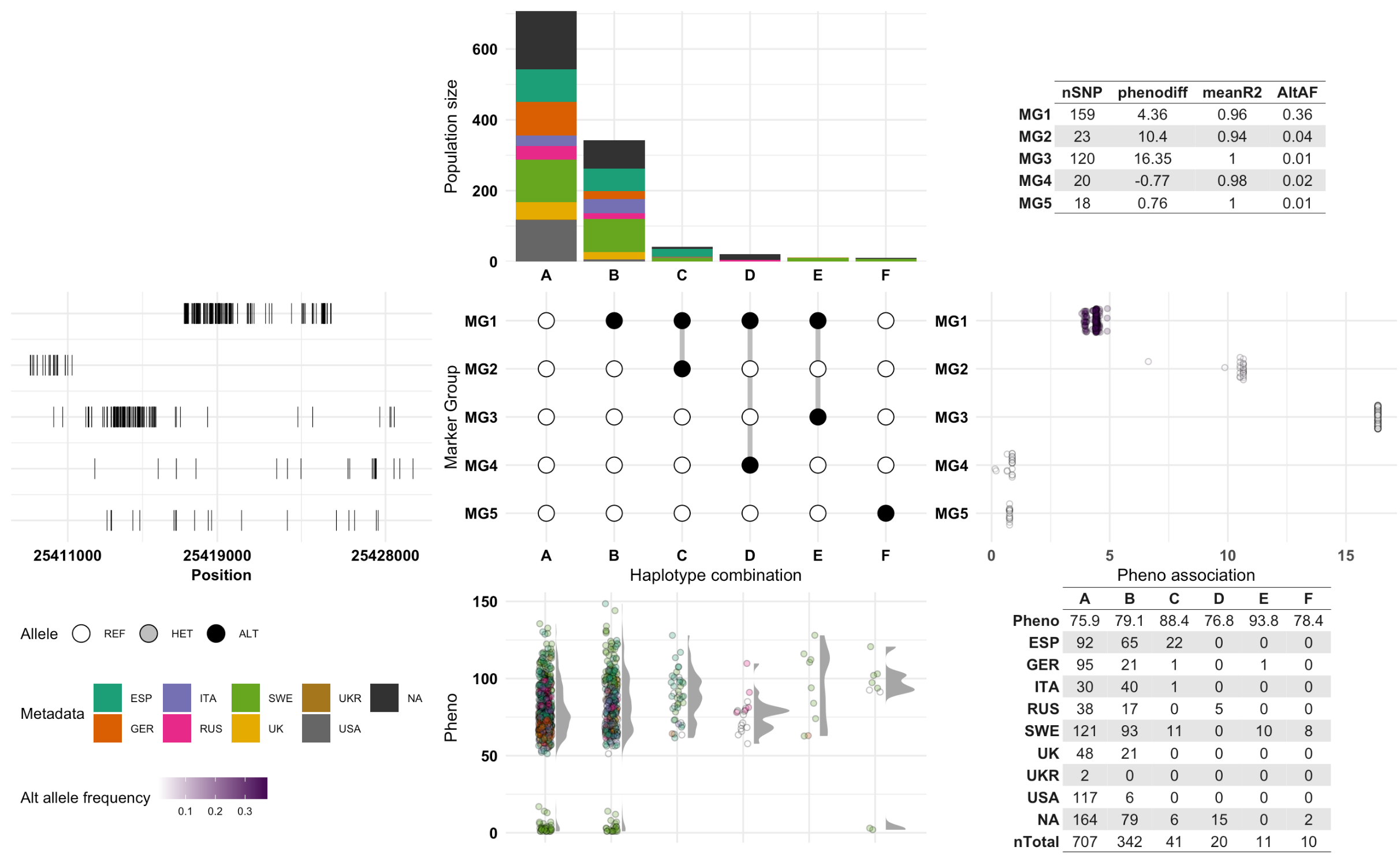


**Supplementary Figure 4.** Crosshap visualization of Chr1@25408933–25429985 at epsilon = 1 using the *A. thaliana* 1001 Genomes data. See <https://jacobimarsh.github.io/crosshap/articles/Getting_started.html#visualizing-local-haplotypes> for details on what each subplot represents, and advice on interpreting the crosshap visualization. The `plot_left = “pos”` option was used with crosshap to display the position of each variant within each Marker Group, instead of the minor allele frequencies.

At epsilon = 1, five Marker Groups (clusters of linked SNPs) were identified, with a range of characteristics. The 159 SNPs in MG1 (for which the alternate allele is present in accessions with haplotypes B-E) have a high alternate allele frequency (0.36), which is typically associated with a mean flowering time 4.36 days later than accessions with reference alleles for MG1 SNPs. MG2 represents 23 SNPs located within a 2.2kb region from 25408991 to 25411231. Alternate alleles for MG2 SNPs are most commonly possessed by Spanish accessions. MG3 represents 120 SNPs in perfect linkage (R^2^ between all SNPs = 1) spanning the majority of the 22kb region. The alternate alleles for MG3 are only possessed by accessions with the E haplotype, who share the alternate alleles for MG1 with haplotypes B-D). Accessions with the alternate alleles for MG3 have a mean flowering time 16.35 days later than accessions with the reference alleles for MG3 SNPs. Furthermore, the E haplotype appears to be specific to accessions sampled from Sweden, except for a single accession from Germany. While out of the scope of this brief exploratory analysis, further inspection of the variants in MG3, and features of the accessions with haplotype E, may reveal candidate variants causal for flowering time variability, and provide insights into the evolutionary history of this genomic interval.

# Supplementary Note 3: Variant dimension reduction clustering algorithms

The aim of variant dimension reduction for local haplotyping is to identify distinct groups of co-inherited variants in high LD with each other that can be represented by single data points, which are termed ‘marker groups’. The fewer the number of groups that variants are clustered into, the fewer the dimensions of variability which in turn improves interpretability of the haplotypes. However, decreasing dimensionality too far comes at the cost of internal marker group homogeneity and leads to highly dissimilar variants being treated as identical in downstream analysis. To maximize the utility of each marker group, crosshap’s variant clustering only retains core groups of variants that are highly internally linked, whilst removing independent variants that are not part of major linkage patterns in the region (see main text).

### Centroid-based clustering

Local haplotyping involves characterizing high-dimensional variant data for which the correct number of groups to assign variants cannot be known or estimated without complete ARG information (local trees needed), especially as overall genealogical relatedness between individuals and populations will not necessarily be directly reflected in local genomic heritability patterns. As a result, traditional centroid-based clustering methods such as K-means and K-mediods that rely on users to define the number of clusters in a dataset *a priori* are not ideally suited for the task of variant clustering (Thalamuthu et al., 2006; Karim et al., 2021). X-means is a centroid-based alternative that overcomes this limitation by automatically determining the number of clusters. However, as with other centroid-based methods, X-means relies on the assumption that there is a central node for each cluster that correlated variables are placed around in a spherical Gaussian distribution (Tomasev et al., 2014). This is inappropriate for clustering variants as complex population structure, selection effects on distal unlinked QTLs and recombination stochasticity are likely to result in linked marker groups that are not spherically distributed around a central point, even if a single variant has experienced direct selection.

### Hierarchical clustering

Hierarchical clustering improves over basic centroid-based methods by aiding the user in selecting the optimal number of clusters (Rokach and Maimon, 2005). However, both hierarchical and centroid-based methods are highly sensitive to outliers, and are not intended to identify outliers, as they primarily work by partitioning all data points based on their relationships with all other data points (Karim et al., 2021). For the purposes of variant dimension reduction, this dilutes the effect of local clusters of densely linked variants, which are the patterns that need to be identified and isolated.

### Density-based clustering

Density-based spatial clustering of applications with noise (DBSCAN) (Ester et al., 1996) is an algorithm implemented for variant dimension reduction in crosshap. DBSCAN is well-suited to high-dimensional variant data and possesses several key advantages over hierarchical and centroid-based clustering for capturing linked marker groups (Karim et al., 2021). Firstly, DBSCAN defines clusters by only considering the density of proximal (correlated) points and classifies points that are not in a dense cluster of points as noise. Therefore, not only does DBSCAN natively bypass the need for applying post-hoc outlier detection algorithms, but more critically, the clusters themselves are defined without being biased by noise in the dataset (Schubert et al., 2017).

DBSCAN ‘builds’ clusters from nodes of dense points by finding connected paths with other densely correlated neighbouring points (Ester et al., 1996). Therefore, DBSCAN does not assume cluster sphericity and can classify marker groups that are irregularly distributed. However, a drawback of this feature is that when large clusters are non-spherical, distant points within the cluster can be unpredictably dissimilar to each other; i.e., there is no theoretical limit to how uncorrelated two points within a cluster can be if there are enough intermediate points ‘connecting’ them. This pitfall is overcome in crosshap by a ‘smoothing’ step during variant dimension reduction that removes outlier points in each cluster.

### Variant cluster smoothing

The cluster ‘smoothing’ is performed by calculating the mean pairwise linkage (R^2^) of each SNP in a cluster with all other points within the same cluster. Loci that are in very high linkage with many other points in the cluster will have a high mean intra-cluster R^2^, whereas outlier loci will have a low mean intra-cluster R^2^. To classify outliers, the standard deviation of the mean intra-cluster R^2^ is calculated, and loci that exhibit a score 2 standard deviations below the median of the mean intra-cluster R^2^ for a given cluster will be removed (Supplementary Figure 5). Cluster ‘smoothing’ ensures that while initial clustering is performed without assumptions regarding the shape of the data, clusters are marginally pruned to ensure that each locus within a marker group is in high linkage with all other loci, removing the number of outlier SNPs (Supplementary Figure 5). The ‘smoothing’ establishes a minimum level of sphericity between SNPs within each marker group to improve internal homogeneity (Supplementary Figure 5B).


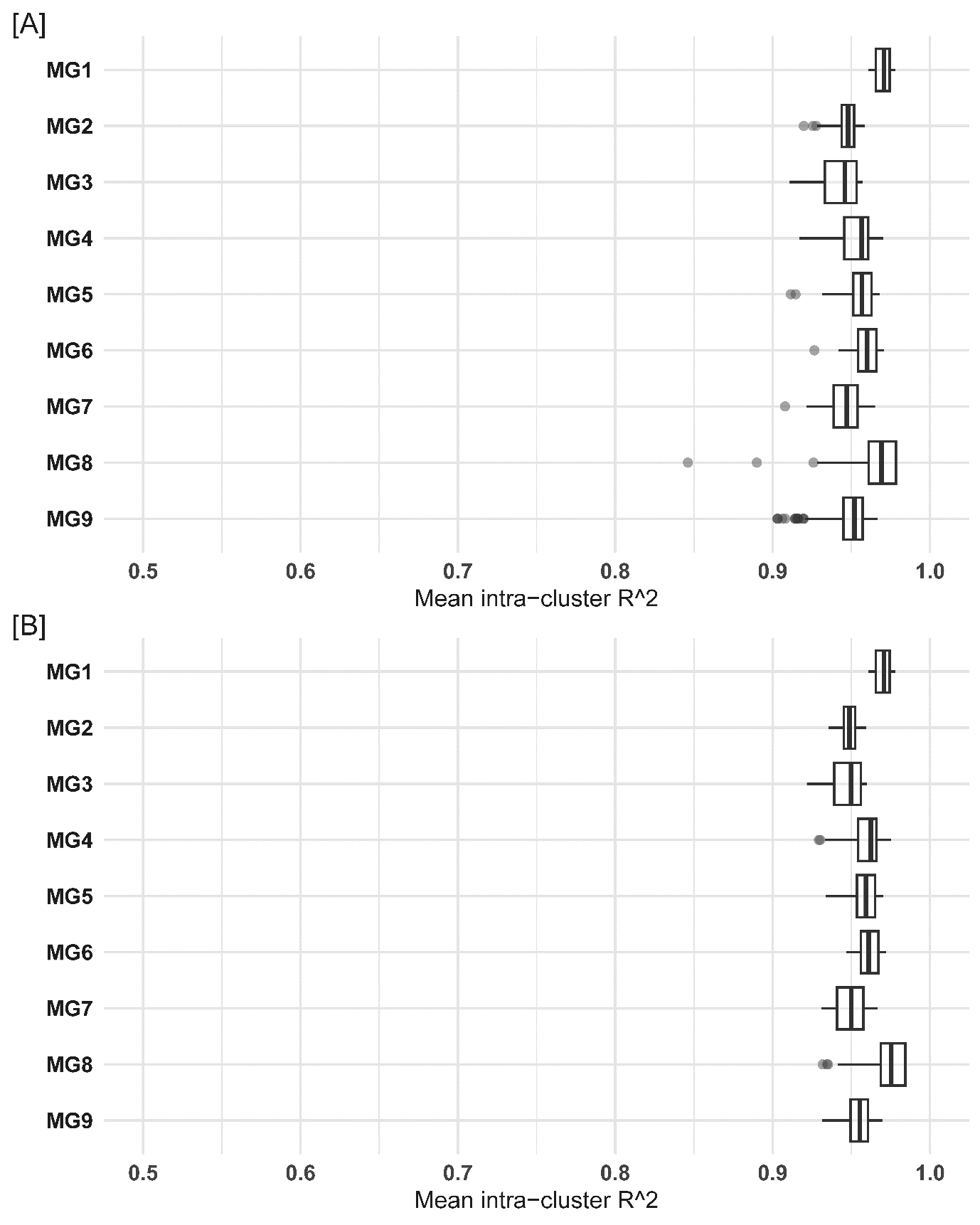


**Supplementary Figure 5.** Boxplot of mean linkage (R^2^) of each SNP with other SNPs within the same marker group; without cluster smoothing **[A]** and after cluster smoothing **[B]**. N.B. outlier points in **[B]** are defined in reference to the mean and standard deviation of the marker groups after smoothing and were not greater than 2 standard deviations below the median in **[A]**.

run_haplotyping(vcf, LD, metadata, pheno, MGmin = 30, keep_outliers = T)

A <- build_right_clusterplot(Haplotypes_MGmin30_E0.6, hide_labels = T

run_haplotyping(vcf, LD, metadata, pheno, MGmin = 30, keep_outliers = F)

B <- build_right_clusterplot(Haplotypes_MGmin30_E0.6, hide_labels = T)

Successful clustering with DBSCAN is highly contingent on the use of appropriate minimum points (minPts) and epsilon parameters. In the context of local haplotyping, minPts may be set based on the minimum number of SNPs the user chooses to allow in each marker group. This may change depending on the density of SNPs captured in the region, though values in the range of 10-30 will typically be appropriate. Epsilon is a statistical term that in the case of DBSCAN, refers to the local radius of points in a cluster within which new points are counted and recursively incorporated (if they exceed the minPts threshold). Choosing an appropriate epsilon value is a major obstacle to the accessibility of local haplotyping using DBSCAN, as even though epsilon roughly translates to ‘cluster density’, it is highly dataset dependent and laborious to optimize (Kriegel et al., 2011).

### Hierarchical density-based clustering

Hierarchical density-based spatial clustering of applications with noise (HDBSCAN) (McInnes et al., 2017) is an extension of DBSCAN which bypasses the need to specify an epsilon parameter. While DBSCAN requires a static epsilon value across an entire dataset, HDBSCAN instead algorithmically optimizes the epsilon parameter separately for each cluster. Therefore, HDBSCAN can capture clusters of varying densities, and only requires a minPts parameter as input. A (now deprecated) HDBSCAN implementation of local haplotyping is available in crosshap and was tested against a carefully chosen DBSCAN epsilon parameter (0.6).


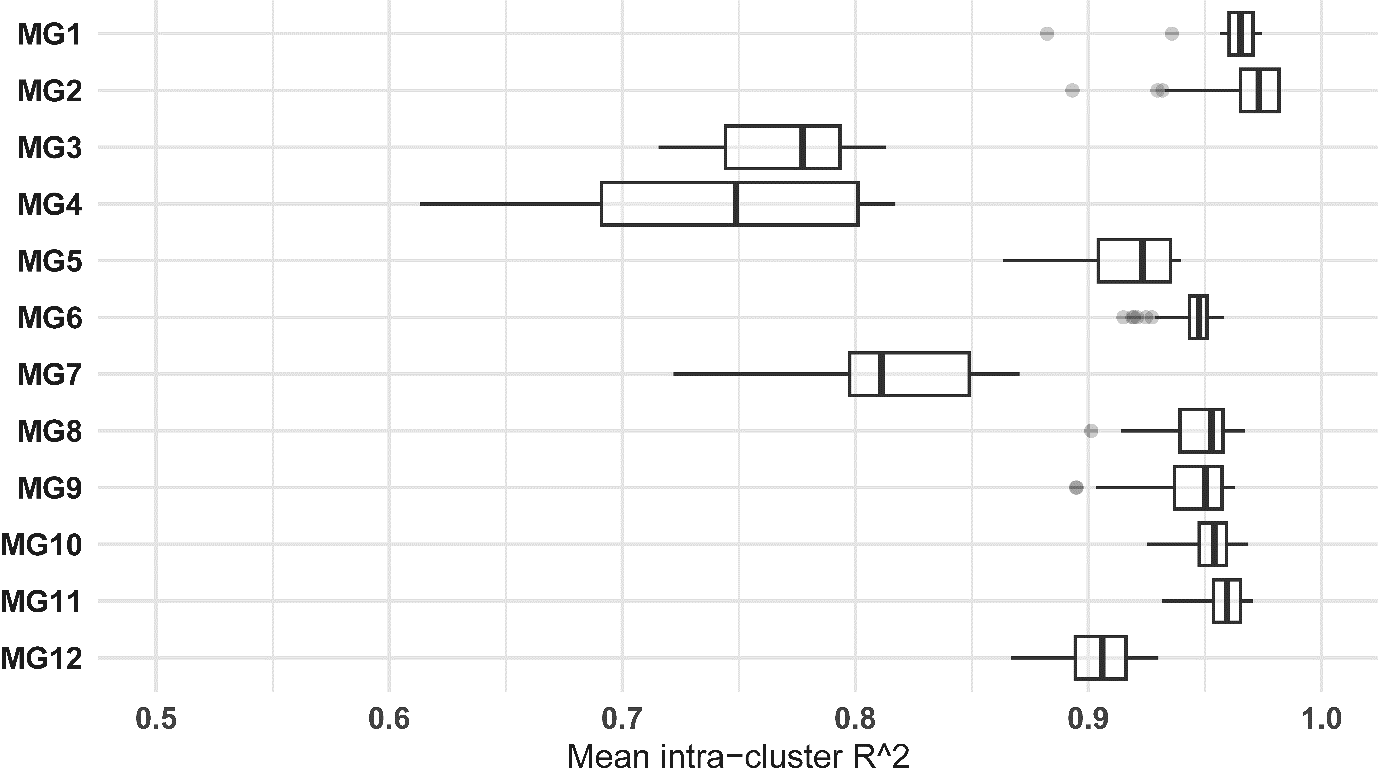


**Supplementary Figure 6.** Boxplot of mean linkage (R^2^) of each SNP with other SNPs within the same marker group defined by HDBSCAN with cluster smoothing.

run_hdbscan_haplotyping(vcf, LD, metadata, pheno, MGmin = 30, keep_outliers = F)

build_right_clusterplot(Haplotypes_MGmin30_HDBSCAN, hide_labels = T)

HDBSCAN identified many marker groups; however, this led to the clustering of marker groups that possessed poor internal linkage (Supplementary Figure 6). Variability in the density of clusters is not necessary for local haplotyping as all marker groups need to be highly internally linked, which can be provided by a single stringent epsilon value. Therefore, DBSCAN was chosen as the default clustering algorithm for variant reduction and tools were developed to aid the user in choosing an epsilon parameter that is optimal for a given dataset (see main text).

# References

**Ester M, Kriegel H-P, Sander J, Xu X** (1996) A density-based algorithm for discovering clusters in large spatial databases with noise. *In* Proceedings of the 2nd ACM International Conference on Knowledge Discovery and Data Mining (KDD), pp 226-231

**Browning BL, Tian X, Zhou Y, Browning SR** (2021) Fast two-stage phasing of large-scale sequence data. Am J Hum Genet 108(10):1880-1890.

**Karim MR, Beyan O, Zappa A, Costa IG, Rebholz-Schuhmann D, Cochez M, Decker S** (2021) Deep learning-based clustering approaches for bioinformatics. Brief Bioinform **22:** 393-415

**Kriegel HP, Kröger P, Sander J, Zimek A** (2011) Density‐based clustering. Wiley Interdiscip Rev: Data Min Knowl Discov **1:** 231-240

**Li X, Shi Z, Gao J, Wang X, Guo K** (2023) CandiHap: a haplotype analysis toolkit for natural variation study. Molecular Breeding **43**

**Marsh JI, Hu H, Petereit J, Bayer PE, Valliyodan B, Batley J, Nguyen HT, Edwards D** (2022) Haplotype mapping uncovers unexplored variation in wild and domesticated soybean at the major protein locus cqProt-003. Theor Appl Genet **135:** 1443-1455

**McInnes L, Healy J, Astels S** (2017) hdbscan: Hierarchical density based clustering. J Open Source Softw **2**

**Purcell S, et al.** (2007) PLINK: A Tool Set for Whole-Genome Association and Population-Based Linkage Analyses. Am J Hum Genet 81(3): 559–575.

**Rokach L, Maimon O** (2005) Clustering Methods. *In* Data Mining and Knowledge Discovery Handbook, pp 321-352

**Schubert E, Sander J, Ester M, Kriegel HP, Xu X** (2017) DBSCAN Revisited, Revisited. ACM Transactions on Database Systems **42:** 1-21

**Tardivel A, Torkamaneh D, Lemay MA, Belzile F, O'Donoughue LS** (2019) A systematic gene‐centric approach to define haplotypes and identify alleles on the basis of dense single nucleotide polymorphism datasets. Plant Genome **12**

**Thalamuthu A, Mukhopadhyay I, Zheng X, Tseng GC** (2006) Evaluation and comparison of gene clustering methods in microarray analysis. Bioinformatics **22:** 2405-2412

**Tomasev N, Radovanovic M, Mladenic D, Ivanovic M** (2014) The Role of Hubness in Clustering High-Dimensional Data. IEEE Transactions on Knowledge and Data Engineering **26:** 739-751

**Wang CC, Yu H, Huang J, Wang WS, Faruquee M, Zhang F, Zhao XQ, Fu BY, Chen K, Zhang HL, Tai SS, Wei C, McNally KL, Alexandrov N, Gao XY, Li J, Li ZK, Xu JL, Zheng TQ** (2019) Towards a deeper haplotype mining of complex traits in rice with RFGB v2.0. Plant Biotechnology Journal **18:** 14-16

**Wu X, Jiang W, Fragoso C, Huang J, Zhou G, Zhao H, Dellaporta S** (2022) Prioritized candidate causal haplotype blocks in plant genome-wide association studies. PLoS Genet **18**
